# Low immunization coverage in Wonago district, southern Ethiopia: A community-based cross-sectional study

**DOI:** 10.1371/journal.pone.0220144

**Published:** 2019-07-24

**Authors:** Samrawit Hailu, Ayalew Astatkie, Kjell Arne Johansson, Bernt Lindtjørn

**Affiliations:** 1 School of Public Health, College of Medicine and Health Sciences, Dilla University, Dilla, Ethiopia; 2 School of Public and Environmental Health, College of Medicine and Health Sciences, Hawassa University, Hawassa, Ethiopia; 3 Centre for International Health, University of Bergen, Bergen, Norway; University of Campania, ITALY

## Abstract

**Introduction:**

Immunization is a cost-effective intervention that prevented more than 5 million deaths worldwide from 2010 to 2015. Despite increased vaccination coverage over the past four decades in many African countries, including Ethiopia, universal coverage has not yet been reached. Only 39% of children aged 12–23 months received full vaccinations in Ethiopia, according to the 2016 Ethiopian Demographic Health Survey. This study aimed to evaluate immunization coverage and identify individual and community factors that explain incomplete vaccination coverage among children aged 6–36 months in the Wonago district of southern Ethiopia.

**Methods:**

We conducted a community-based, cross-sectional study in three randomly selected kebeles in the Wonago district from June to July 2017. Our nested sample of 1,116 children aged 6–36 months included 923 child-mother pairs (level 1) within kebeles (level 2). We conducted multilevel regression analysis using STATA software.

**Results:**

Among participants, 85.0% of children aged 12–36 months received at least one vaccine, and 52.4% had complete immunization coverage. After controlling for several individual and community variables, we identified six significant predictor variables for complete immunization: Older mothers’ age (AOR = 1.05, 95% CI: 1.00–1.09), higher utilization of antenatal care (AOR = 1.36, 95% CI: 1.14–1.62), one or more tetanus-toxoid vaccination during pregnancy (AOR = 2.64, 95% CI: 1.43–4.86), mothers knowing the age at which to complete child’s vaccinations (AOR = 2.00, 95% CI: 1.25–3.20), being a female (AOR = 0.64, 95% CI: 0.43–0.95), and child receiving vitamin A supplementation within the last 6 months (AOR = 2.79, 95% CI: 1.59–4.90). We observed a clustering effect at the individual and community levels with an intra-cluster correlation coefficient of 48.1%.

**Conclusions:**

We found low immunization coverage among children in the Wonago district of southern Ethiopia, with significant differences across communities. Promoting maternal health care and community service could enhance immunization coverage.

## Introduction

Immunization is a cost–effective and lifesaving intervention that prevents sickness, disability, and death. Between 2010 and 2015, immunizations prevented more than 5 million deaths worldwide [1, 2]. In Ethiopia, vaccination programs averted 23% of deaths among children [3]. Vaccination coverage is an important indicator to monitor progress towards the United Nation’s Sustainable Development Goal 3 “Ensure healthy lives and promote well-being for all at all ages” [4, 5]. Specifically, this goal aims to eradicate preventable newborn and under-five mortality by 2030 and reduce under-five mortality to 25 per 1,000 live births [4, 5].

The World Health Organization (WHO) launched the global Expanded Program on Immunization (EPI) in 1974 to control and reduce vaccine-preventable disease and death among children throughout the world. For children under one year old, the program recommends country-level immunization rates of 90% and at least 80% within districts or equivalent administrative units [6–9]. From 2010 to 2016, global immunization coverage stalled at 86%. Worldwide, vaccine coverage remained high, but regional rates varied, particularly among children in low- and middle-income countries, such as sub-Saharan Africa [10, 11]. For example, using the diphtheria-pertussis-tetanus-3 vaccine as a key indicator (because it tends to largely be distributed through horizontal health programs and not vaccine campaigns such as supplementary immunization activities, it is the proxy for the completion of vaccination series and ability health system to reach children multiple times), the coverage among children younger than 1 year was 86% globally, 75% in Africa, and 96% in the western Pacific and Europe. Most children (14.8 million;68%) who did not receive this vaccine lived in 10 countries, including Ethiopia [12].

Ethiopia has a high burden of diarrheal diseases, vaccine-preventable diseases, and malnutrition [13]. In 1980, the Ethiopian Ministry of Health launched its own immunization program to increase vaccine coverage by 10% annually and reach 100% coverage among children younger than 2 years by 1990 [14]. These implementation guidelines were revised in 2015 to focus on children younger than one year and women of reproductive age (15–49 years) as the main targets for currently available vaccines in Ethiopia. Progress has been made [7]. Diphtheria-pertussis-tetanus-3 coverage reached 3% in 1980 and 49% in 1990 [15]. This coverage has gradually increased with the Reach Every District initiative, started in 2004, and the health extension program, implemented in 2003 [16]. Unfortunately, full immunization coverage has not been reached. The 2016 Ethiopian Demographic Health Survey reported only 39% vaccination rates in children aged 12–23 months and that rate varied substantially across geographical regions. For example, coverage in the Southern Nation, Nationalities, and Peoples’ Region was 47% [17]. Despite outreach strategies and supplementary immunization schedules, full coverage remains very low in some regions of Ethiopia (e.g. Afar and Somali).

To achieve the goal and reduce child mortality, vaccinating children should be maintained. So, understanding the level and factors that affect the coverage should be known. Previous studies evaluate the coverage and suggest that antenatal care, mothers’ tetanus vaccination status, place of birth, mother’s education, mother’s knowledge about vaccination, and household wealth can affect immunization coverage [18–27]. However, these studies focus on individual factors while neglecting community, household, and institutional factors. The objective of this study thus was to evaluate immunization coverage and to identify factors associated vaccination coverage at individual and community levels among children aged 6–36 months. Children age less than 11 months were focused to see the immunization coverage of children assessed for child immunized accordance to their age. We conducted this study in the Gedeo Zone of southern Ethiopia. The results would help policymakers, program implementers, and service providers to address issues that decrease vaccination rates.

## Materials and methods

### Ethical clearance

The institutional review board at the College of Medicine and Health Sciences of Hawassa University (reference number: HW/17/0668/15) and the regional ethical committee in Western Norway (reference number: 2016/1916/REK vest) provided ethical approval. Permission letters were obtained from Gedeo zone health department and Wonago district health office. Before the start of the study, community elders, health extension workers and kebele leaders were sensitized. Mothers or caretakers provided written and signed informed consent. Confidentiality was maintained and participants were informed that participation was voluntary and they had right to withdraw from the study at any time.

### Study setting and design

We conducted community-based, cross-sectional study in Wonago district in southern Ethiopia from June 2017 to July 2017. Wonago is about 377 km south of Addis Ababa and 13 km south of Dilla, the capital of Gedeo. The district is 142 km^2^ and has 17 rural and 4 urban kebeles (the smallest administrative units) containing 29,227 households. Among the most densely populated areas in Ethiopia, it has 1,014 people per square kilometer. According to the 2007 census, the district’s population of 147,600 people included 22,140 (15%) children younger than five years [28, 29]. The majority of the population lives in the rural areas and agricultural is the dominant means of livelihood of Wonago district. Major cause of childhood illness is pneumonia and diarrhea. Wonago has 20 health posts, six governmental health center, two private clinics, and two drug stores. Expanded Program of Immunization (EPI) was all provided in health centers and health posts and supported by 34 outreach programs site with every week provision of immunization.

### Study participants

Eligible participants included were all children aged 6 to 36 months and their mothers or guardians, who lived in Wonago for at least 6 months before data collection and who consented to participate in the study. We excluded those who had not lived in the study area for least 6 months prior to data collection. We calculated the sample size required for estimating immunization coverage using Open Epi software version 3.03 [30]. The calculation assumed a desired precision (sampling error) of 4% to get larger sample size with a 95% confidence interval (CI), a design effect of two to consider two stage sampling and to adjust the variance, and a 10% non-response rate. The anticipated proportion of full immunization coverage was 30.5% based on a study in Hosanna [20]. We thus calculated a sample size of 1,119 children aged 6 to 36 months old and their guardians.

A two-stage sampling technique was employed. In the first stage, we used a random sampling lottery method to select 3 of 17 total kebeles using the Statistical Package for Social Science (SPSS) version 20 complex sample method [31], we then randomly selected 12 villages from the selected kebeles based on probability proportional to size (number of households). Before the survey, we conducted a census in selected kebeles to obtain socio-demographic, household status information and to identify eligible children. The sample is distributed to selected villages based on probability proportional to size (number of eligible children). The first household was identified by randomly from the center of the village. Once first household was identified the interviewer went to the next household with the mother of children age group of 6–36 months. Subsequent sampling of household was conducted from selected villages until the desired sample size was attained. When two children from the same household were identified, both children were selected.

### Study variables

The outcome variable in this study was full (complete) immunization. Individual-level factors included were mother’s age, mother’s occupation, parity, religion, ethnicity, women’s education(Mothers education has 5 categories, no formal education, primary(1–8 grade), secondary(9–10 grade), preparatory (11–12 grade) and college or university), antenatal care (antenatal care defines as number of visit that mother get care during pregnancy), delivery place, post-natal care, sex of child, number of children younger than five years in the household, birth order, family planning use, household family size, attitude of mothers towards immunization(Mothers attitude towards vaccination was assessed by six attitude related questions and using a 2-point scale (agree and disagree), where 1 = positive perception and 0 = negative perception. The mean score was computed and dichotomized into positive and negative: if mothers reacted score below the mean, they were labeled as having a negative attitude; if mother reacted to at the mean and above the mean, they were labeled as having a positive attitude), presence of child vaccination card and wealth index. The wealth index was assessed to capture households’ socio-economic statuses and constructed using principal component analysis based on household asset and amenities. The generated wealth score was grouped into quartiles as a measure of socioeconomic status, with the first quartile representing the poorest group and the fourth quartile the richest.

Community-level factors included were visits from healthcare workers, distance to healthcare facilities, access to health outreach (e.g. vitamin A supplementation) and families participating in food supporting programs(such as safety net program).

Table 1 summarizes the infant immunization schedule of recommended vaccines, including Bacillus Calmette–Guérin; oral polio; diphtheria, pertussis, and tetanus; hepatitis B and *Hemophilus influenzae* type B; pneumococcal conjugate; rotavirus; and measles) in the study area [14].

**Table 1:** National vaccine schedule for infants in Ethiopia.

Age	Visit	Antigen
**Birth**	1	Bacillus Calmette–Guérin (BCG) Oral polio #0 (Polio0)
**6 weeks**	2	Diphtheria, pertussis, & tetanus Hepatitis B #1 and *Hemophilus influenzae* type B #1(Pentavalent1) Oral polio #1 (Polio1) Pneumococcal conjugate #1(PCV1) Rotavirus #1(Rota1)
**10 weeks**	3	Diphtheria, pertussis, & tetanus Hepatitis B #2 and *Hemophilus influenzae* type B #2(Pentavalent2) Oral polio #2 (Polio2) Pneumococcal conjugate #2 (PCV2) Rotavirus #2 (Rota2)
**14 weeks**	4	Diphtheria, pertussis, & tetanus Hepatitis B #3 and *Hemophilus influenzae* type B #3(Pentavalent3) Oral polio #3 (Polio3) Pneumococcal conjugate #3 (PCV3)
**9 months**	5	Measles

### Data collection and quality control

The structured questionnaire was initially developed in English, translated into the local Gedeoffa language and then translated back to English to ensure consistency. Most questions were adopted from questionnaires in the Demographic and Health Survey of Ethiopia [5] and from literature reviews [15, 21, 23–25]. Before data collection the questionnaires reviewed by supervisors then we conducted pre-test on 56 children (6–36 months) in another kebele of Wonago district and that were not included in the study. Based on this pre-testing, we rephrased unclear questions, wording, and sequences. After data collection the data cleaned and checked for the consistency.

Eight trained data collectors and two supervisors conducted the interviews. Immunization data were collected from vaccination cards (i.e., doses and types) and mothers’ or guardians’ verbal reports. We confirmed the information given by checking children for Bacillus Calmette–Guérin (BCG) scars on upper arm. The principal investigator checked the data for completeness, and errors were corrected accordingly. To control for recall bias, we used standardized questionnaires and trained data collectors in facilitating participant recall.

We defined complete or full immunization among children aged 12–36 months as receiving one dose of BCG, three doses of polio, three doses of (Diphtheria, pertussis, tetanus, Hepatitis B and *Hemophilus influenzae* type B) and one dose of measles, as confirmed by immunization card or mother’s recall. We defined partial immunization as missing one or more of the recommended eight vaccines and children who were not vaccinated at recommended age. Children who never received any immunizations were classified as not vaccinated. We considered children younger than 11 months with complete vaccinations as completed immunization for age. We defined the age limit for immunization as nine months, by which each child should have had one dose of BCG, three doses of polio, three doses of pentavalent, and one dose of measles vaccinations. Immunization coverage by card was calculated based on card documentation only and excluded vaccinations confirmed by mothers’ recall. Immunization by card plus recall included card and verbal histories. An infant immunization card was yellow card that given to child when child starts vaccination and used for vaccination follow-up and monitoring.

### Data analysis

Data were double-entered using EpiData version 3.1. STATA 15 software (Stata Corp) was used for analyses. We compiled descriptive statistics, such as frequencies, percentages, means, and ranges. Cross-tabulation was used to show proportions of different categories of each characteristic, with respect to immunization status. A two-level logistic regression model was applied to analyze the hierarchical structure. Child-mother pairs (level 1) were nested within communities or villages (level 2).

We used multilevel analysis to account for hierarchical and binary outcome variables. Four models were constructed. The first (null) model had no exposure or independent variables and was used to check there was variability in probability of children with fully immunized across the community. The second and third models comprised individual and community variables, respectively. The fourth multivariate, multilevel regression model adjusted for outcome variables and predictors that were significant at the individual or community level. The effects of individual and community level predictors on the dependent variable were assessed independently. Bivariate analysis was performed to test the effect of each independent variable on the immunization coverage. Only variables correlating with fully immunization (for our data set defined as all variables correlating with immunization with p-value of ≤ 0.25) were selected for the consecutive multivariate analysis[32]. Multicollinearity testing was performed using Variance Inflation Factors (VIF) and independent variables with VIF >5 were removed.

Estimated associations (fixed-effects) between the likelihood of full vaccination and various explanatory variables were expressed as adjusted odds ratios (AOR) with 95% CI. Variations (random effects) were reported as intra-cluster correlation coefficients, or the percentage of variance explained by the community-level variables [33].

The Akakie information criterion was used to estimate goodness-of-fit of the adjusted final model in comparison with the preceding individual- and community-level models. The model with the lowest value was considered the best-fit model [33]. All variables with P-values less than 0.25 in the bivariate analysis were included in the multivariate logistic regression.

## Results

Among the 923 mothers who participated in this study, the mean age was 27.4 (15–50) years, 798 (86.7%) were housewives and 674 (73.0%) had no formal education. Nearly all (909; 98.5%) were married. More than half (564; 54.6%) had households of 6 or more people. Only 339 (43.2%) of households had been visited by health extension workers in the past month. Table 2 summarizes the results for the mothers and guardians who participated in the study.

**Table 2:** Socio-demographic characteristics of mothers in the Wonago district, Gedeo zone, in Southern Ethiopia, 2017 (n = 923).

Characteristics of mothers	Frequency	Percent (%)
**Age (years)**		
<25	380	41.0
26–34	432	46.8
35–45	106	11.5
>46	5	0.5
**Formal education**		
None	674	73.0
Primary level	220	23.8
Secondary and above	29	3.1
**Marital status**		
Married	909	98.2
Divorced/widowed	14	1.5
**Occupation**		
Housewife	798	86.7
Merchant	72	7.8
Other	53	5.7
**Religion**		
Protestant	860	93.2
Orthodox	45	4.8
Other religion	18	2
**Family size**		
≤5	419	45.4
≥6	504	54.6
**Travel time to health post**		
<15 minutes	232	25.1
15–29 minutes	134	13.9
30–60 minutes	294	31.9
≥60 minutes	263	28.5
**Wealth quartile**		
Poorest	220	23.8
Poor	237	25.7
Medium	233	25.2
Rich	233	25.2
**Food safety net program participant**		
No	768	83.2
Yes	155	16.8
**Health extension worker visit in last month**		
No	524	56.8
Yes	399	43.2

Of the 1,116 (571 girls and 545 boys) children aged 6–36 months in this study, the mean age was 24.1 months. Most (799; 71.3%) were born at home, and 317 (28.7%) were born at a health institution. Mothers reported that 178 (15.9%) experienced illness in the two weeks preceding the survey. Table 3 summarizes the results for the children.

**Table 3:** Socio-demographic characteristics of children aged 6–36 months in the Wonago district, Gedeo zone, Southern Ethiopia, 2017 (n = 1,116).

Characteristics of children	Frequency	Percent (%)
**Sex**		
Male	571	51.2
Female	545	48.8
**Age (months)**		
6–11	131	11.7
12–23	274	24.6
24–36	711	63.7
**Birth order**		
First	185	16.6
Second	183	16.4
Third to fifth	511	45.8
Sixth and above	237	21.2
**Place of delivery**		
Home	799	71.6
Health institution	317	28.4
**Illness in past 2 weeks**		
No	938	84.1
Yes	178	15.9

### Maternal health care

Most (863; 77.3%) mothers had at least one antenatal care visit during pregnancy, 351 (40.6%) had three antenatal care visits, and 246 (28.5%) had four and more. More than half (766; 68.6%) were immunized against tetanus, and 766 (68.6%) received no postnatal care for the child who participated in the study. Table 4 summarizes the results for maternal health care.

**Table 4:** Maternal health care in the Wonago district, Gedeo zone, Southern Ethiopia, 2017.

Maternal health variables	Frequency	Percent (%)
**Antenatal care (n = 1,116)**		
Yes	863	77.3
No	253	22.7
**# antenatal care visits (n = 863)**		
1	20	2.4
2	129	15.0
3	351	40.6
>4	246	28.5
Do not remember	117	13.5
**1 or more tetanus vaccines**		
No	350	31.4
Yes	766	68.6
**Prenatal care**		
No	766	68.6
Yes	350	31.4

### Awareness of vaccinations among mothers and guardians

Of the 879 (94.7%) mothers who knew about vaccinations, 604 (68.0%) got the information from health workers and 128 (14.0%) from community volunteers. Only 130 (14.1%) women knew that vaccinations start at birth, but more than half (593; 64.2%) knew the age at which a child should complete immunization. Table 5 summarizes the results.

**Table 5:** Mother's awareness of vaccinations in the Wonago district, Gedeo zone, in Southern Ethiopia, 2017 (n = 923).

Mothers’ vaccine knowledge	Frequency	Percent (%)
Knew about vaccines	879	95.2
Did not know about vaccines	44	4.8
Source of information (n = 879)*		
Health worker	604	68.7
Radio	12	1.4
Television	10	1.1
Friends	41	4.7
School	27	3.1
Neighborhood	56	6.1
Community volunteer	128	14.6
**Age to start vaccinations**		
Birth	130	14.1
1 month	716	77.6
Do not know	77	8.3
# vaccination sessions needed*		
1	8	0.9
2	20	2.2
3	222	24.1
4	326	35.3
5	205	22.2
Do not know	135	14.6
**Age to complete vaccinations**		
Before 1 year or 9 months	593	64.2
1 year or older	191	20.7
Do not know	138	15.0

*The variables that had missing data result from incomplete recording

### Immunization coverage

Based on immunization cards and mothers’ recall, 959 (85.9%) children had at least one vaccine dose (95% CI; 83.9, 87.9). Among vaccinated children, 585 (52.4%) were fully immunized, 333 (29.8%) were partially immunized, 158 (14.2%) were not immunized, and 40 (3.6%) had completed immunization-for-age at the time of the survey. Among fully vaccinated children, (151) 55.1% were 12–23 months old, and (434) 60.9% were 24–36 months old. Table 6 summarizes the results.

**Table 6:** Age-distributed immunization status of children in the Wonago district, Gedeo zone, in Southern Ethiopia, 2017.

Age of child (months)	Immunization status of children			
	Fully immunized	Completed for age	Partially immunized	Not immunized
**6–11**	not applicable	40 (30.5%)	59 (45.0%)	32 (24.4%)
**12–23**	151 (55.1%)	not applicable	87 (31.8%)	36 (13.1%)
**24–36**	434 (60.9%)	not applicable	187 (26.3%)	90 (12.7%)

Only 202 (21.1%) children had vaccination cards, among whom 158 (78%) received the BCG vaccine. More children (200; 99%) received pentavalent #1 than pentavalent #3 (157; 77.7%). Oral polio vaccine #1 was recorded for 200 (99%) of children and oral polio vaccine #3 for 157 (77.7%). Measles vaccines were recorded for 102 (50.4%) children. The lowest coverage was for the oral polio vaccine #0 vaccine 16 (7.9%). Table 7 summarizes the results.

**Table 7:** Vaccination coverage among children aged 6–36 months based on immunization cards, Wonago district, Gedeo zone, Southern Ethiopia, 2017 (n = 202).

Vaccine	Frequency	Percent (%)
Bacillus Calmette–Guérin	158	78.0
Oral polio vaccine #0	16	7.9
Oral polio vaccine #1	200	99.0
Oral polio vaccine #2	181	89.6
Oral polio vaccine #3	157	77.7
Pentavalent #1	200	99.0
Pentavalent #2	185	91.5
Pentavalent #3	157	77.7
Pneumococcal conjugate vaccine #1	192	95.0
Pneumococcal conjugate vaccine #2	178	88.1
Pneumococcal conjugate vaccine #3	151	74.7
Rotavirus #1	191	94.5
Rotavirus #2	165	81.6
Measles	102	50.4

Among partially and unimmunized children, 198 (45.6%) mothers were unaware of the need for subsequent doses of vaccine, 111 (25.0%) were unaware of any need for immunization, and 100 (23.0%) could not remember the place or time of vaccination. Table 8 summarizes the results.

**Table 8:** Reasons for children not being fully immunized in the Wonago district, Gedeo zone, Southern Ethiopia, 2017 (n = 434).

Reasons for partial immunization	Frequency	Percent (%)
Unaware of need for immunization	111	25.6
Unaware of need for subsequent doses	198	45.6
Unknown place and time of immunization	100	23.0
Fear of side effects	52	12.0
Wrong ideas about contraindications	15	3.5
No faith in immunization	7	1.6
Vaccination site was far	9	2.1
Time of vaccination was not convenient	50	11.5
Vaccinator was absent	4	0.9
Vaccine was not available	9	2.1
Child was sick	16	3.7
Long wait	3	0.7

### Bivariate multilevel analysis

In the bivariate analysis, correlation was computed between independent and dependent variables. Maternal age, maternal education and occupation, maternal tetanus immunization, maternal antenatal and prenatal care, sex of child, child birth place, and maternal knowledge about appropriate age to vaccinate, maternal attitude towards immunization, child immunization card, health extension worker visit, and vitamin A supplementation in the last six months were associated with complete immunization. Table 9 summarizes the bivariate analyses.

**Table 9:** Child full immunization status across independent variables, Wonago district, Gedeo zone, Southern Ethiopia, 2017.

Variables	Immunization status		Crude odd ratio(COR)	P-value
	Fully vaccinated # (%)	Not fully vaccinated # (%)		
**Individual factors**				
**Mother’s age (years)**				
Age in years			1.05(1.01–1.09	0.014
**Mother’s occupation**				
Housewife	485 (50.1%)	483 (49.9%)	1	
Merchant	64 (73.6%)	23 (26.4%)	3.88 (1.82–8.28)	<0.001
Other	36 (59.0%)	25 (41.0%)	1.47 (0.60,3.28)	
**Mother’s formal education**				
None	410 (50.2%)	407 (49.8%)	1	
Primary level	155 (58.1%)	112 (41.9%)	1.65 (1.05–2.59)	0.03
Secondary and above	20 (62.5%)	12 (37.5%)	1.84 (0.60–5.60)	
**Mother received 1 or more tetanus vaccines**				
No	113 (32.3%)	237 (67.7%)	1	
Yes	472 (61.6%)	294 (38.4%)	5.09 (3.04–8.52)	0.01
**# antenatal care visits**				
Number of visits	-	-	1.35 (1.15–1.58)	<0.001
**Place of delivery**				
Home	400 (50.1%)	399 (49.9%)	1	
Health institution	185 (58.4%)	132 (41.6%)	1.52 (1.01–2.28)	0.042
**Prenatal care**				
No	381 (59.7%)	386 (50.3%)	1	
Yes	204 (58.3%)	146 (41.7%)	1.7 (1.13–2.58)	0.01
**Age mother reported to complete child’s vaccinations**				
After 1 year	154 (37.7%)	254 (62.3%)	1	
Before 1 year	431 (60.9%)	277 (39.1%)	3.5 (2.21–5.53)	<0.001
**Mother’s attitude towards vaccination**				
Poor	180 (43.8%)	231 (56.2%)	1	
Good	405 (57.4%)	300 (42.6%)	2.09 (1.39–3.15)	<0.001
**Sex of child**				
Female	320 (56%)	251 (44.0%)	1	
Male	256 (48.6%)	280 (51.4%)	0.58 (0.40–0.86)	0.006
**Immunization card**				
No	457 (50.0%)	457 (50.0%)	1	
Yes	128 (63.4%)	74 (36.6%)	1.74 (1.12–2.69)	0.013
**Community factors**				
**Visit from health extension worker**				
No	308 (48.1%)	332 (51.9%	1	
Yes	277 (58.2%)	199 (41.8%)	1.86 (1.26–2.74)	0.002
**Vitamin A supplement**				
No	80 (30.3%)	184 (69.7%)	1	
Yes	505 (59.3%)	347 (40.7%)	6.25 (3.5–11.15)	<0.001

### Measuring variation and association

The null model showed significant variability in the probability of children with complete immunization across kebeles (variance = 2.71, p < .005). The intra-cluster correlation (48.1%) of the variability in probability of complete immunization was related to kebele. Variation in complete immunization across models 1, 2, and 3 was significant. Variation in full immunization in model 4 remained significant (variance = 1.66, p < .001), with 37% of variance among observations attributed to community-level factors.

Table 10 summarizes the multilevel, multivariate logistic regression analysis. After adjusting for variables at the community and individual levels, we found that increase in maternal age corresponded with increase immunization coverage (AOR = 1.05, 95% CI: 1.00–1.09). Children born to mothers who received one or more doses of tetanus toxoid vaccine had significantly higher odds of being fully immunized (AOR = 2.64, 95% CI: 1.43–4.86). Child had higher odds of being fully immunized if their mothers had higher number antenatal care visits (AOR = 1.36, 95% CI: 1.14–1.62). Among children with mothers who knew the age at which a child should complete immunization, 60.9% were fully immunized (AOR = 2.00, 95% CI: 1.25–3.20). Full immunization status of children was higher (60%) among female children than males (AOR = 0.64, 95% CI: 0.43–0.95). Children who received vitamin A supplements in the last 6 months were more likely to be fully immunized (AOR = 2.79, 95% CI: 1.59–4.90).

**Table 10:** Factors associated with full immunization identified by multi-level multivariate logistic regression model, Wonago district, Gedeo zone, Southern Ethiopia, 2017.

Variables	Immunization status		Model 1 (null)	Model 2 AOR (95% CI)	Model 3 AOR (95% CI)	Model 4 AOR (95% CI)
	Fully vaccinated # (%)	Partially vaccinated # (%)				
**Individual**						
**Mother’s age (years)**						
Age in years	-	-	-	1.05 (1.00–1.09)	-	1.05 (1.00–1.10)*
**Mother’s occupation**			-		-	
Housewife	485 (50.1%)	483 (49.9%)	-	1	-	1
Merchant	64 (73.6%)	23 (26.4%)	-	2.13 (0.95–4.77)	-	2.01 (0.86–4.66)
Other	36 (59.0%)	25 (41.0%)	-	1.11 (0.51–2.44)	-	1.03 (0.44–2.31)
**Mother’s formal education**					-	
None	410 (50.2%)	407 (49.8%)	-	1	-	1
Primary level	155 (58.1%)	112 (41.9%)	-	1.09 (0.69–1.61)	-	1.08 (0.67–1.72)
Secondary and above	20 (62.5%)	12 (37.5%)	-	1.24 (0.41–3.77)	-	1.19 (0.37–3.83)
**Mother received 1 or more tetanus vaccines**						
No	113 (32.3%)	237 (67.7%)	-	1	-	1
Yes	472 (61.6%)	294 (38.4%)	-	2.97 (1.64–5.34)	-	2.64 (1.41–4.86)*
**# antenatal care visits**					-	
Number of visits	-	-	-	1.33 (1.13–1.58)	-	1.36 (1.14–1.62)
**Place of delivery**					-	
Home	400 (50.1%)	399 (49.9%)	-	1	-	1
Health institution	185 (58.4%)	132 (41.6%)	-	0.93 (0.62–1.41)	-	1.01 (0.66–1.56)
**Received prenatal care**						
No	381 (59.7%)	386 (50.3%)	-	1	-	1
Yes	204 (58.3%)	146 (41.7%)	-	1.42 (0.93–2.16)	-	1.21 (0.75–1.95)
**Mother’s reported age to complete child vaccinations**						
After 1 year	154 (37.7%)	254 (62.3%)	-	1	-	1
Before 1 year	431 (60.9%)	277 (39.1%)	-	1.92 (1.23–2.99)	-	2.00 (1.25–3.20)*
**Mother’s attitude towards vaccination**			-		-	
Poor	180 (43.8%)	231 (56.2%)	-	1	-	1
Good	405 (57.4%)	300 (42.6%)	-	1.39 (0.93–2.08)	-	1.36 (0.89–2.08)
**Sex of child**						
Female	320 (56.0%)	251 (44.0%)	-	1	-	
Male	256 (48.6%)	280 (51.4%)	-	0.65 (0.45–0.96)	-	0.64 (0.43–0.95)*
**Immunization card**			-		-	
No	457 (50.0%)	457 (50.0%)	-	1	-	1
Yes	128 (63.4%)	74 (36.6%)	-	1.19 (0.75–1.88)	-	1.17 (0.73–1.90)
**Community**						
**Visit from health extension worker**						
No	308 (48.1%)	332 (51.9%)	-	-	1	1
Yes	277 (58.2%)	199 (41.8%)	-	-	1.58 (1.06–2.3)	1.17 (0.75–1.80)
**Vitamin A supplement**						
No	80 (30.3%)	184 (69.7%)	-	-	1	1
Yes	505 (59.3%)	347 (40.7%)	-	-	5.8 (3.28–10.3)	2.79 (1.59–4.90)**
**Random effects**						
Community variance (standard error)			2.71 (1.03)	1.41 (0.88)	2.7 (1.1)	1.66 (0.99)
Intra-cluster correlation			48.10%	32.40%	49.00%	36.70%
**Model fit statistics**						
Log-likelihood			-746	-533	-710	-523
Akakie information criterion			1498	1099	1430	1086

AOR = adjusted odds ratio, CI = confidence interval; Model 1 is an empty, baseline model with no independent variable. Model 2 is adjusted for individual factors. Model 3 is adjusted for community. Model 4 is adjusted for individual and community factors.

*p< 0.05

** p< 0.001

## Discussion

In examining immunization coverage based on individual and community-level characteristics in the Gedeo zone in southern Ethiopia, we found that 85.9% of children had at least one dose of vaccine, but full immunization coverage was low at 52.4%. Immunization coverage was associated with individual factors (e.g., maternal age, prenatal care, mother’s tetanus vaccination, mother’s knowledge about age to complete childhood immunizations, sex of the child), as well as community factors (e.g., vitamin A supplementation). We conducted a two-level, multivariate logistic regression of fixed effects of individual and community factors to show the association of predictors with fully immunization and random effects to explain between-cluster variations. The intra-cluster correlation coefficient was 48.1%, indicating a cluster effect at the individual and community levels. These means the same kebeles were likely to have the same or similar immunization statuses.

Our findings on immunization coverage are comparable to other studies of rural districts in Tigray, Bangladesh, Uganda, and India [34–36]. However, our results show lower coverage than that in other areas of Ethiopia, such as 73% in Arba Minch, 76% in North Gondar, 89% in Ghana, and 86% in Cameroon [18, 19, 22, 37]. The immunization coverage observed in study was higher than result obtained from the report of national demographic health survey (EDHS 2016) 39%, study conducted in Somalia Jijiga 36.5%, in Ambo 36% and in Hossanna 30% [20, 21, 38, 39].

For individual vaccines, 78% of participants in our study received the BCG, 99% received pentavalent #1, 77% received pentavalent #3, and 50% received the measles vaccine. Coverage decreased from the first to final vaccine doses (e.g., oral polio #1 to #3, pentavalent #1 to #3, BCG to measles). This decrease aligns with findings from Arbaminch, Hossana, Ambo, and Bale [19–21, 24]. The long intervals between the third dose of pentavalent and measles vaccines may contribute to low immunization coverage.

In our study, 25% of the children were partially immunized and 14% were never immunized. The main reasons for partial immunization was lack of knowledge among mothers and guardians about the need to return for subsequent doses and the need for immunization in general, as demonstrated by studies in Tigray [32]. Studies conducted in Ghana and Mali also show that insufficient information and inconvenience led to partial immunization among children [18, 40]. It also showed that, fear of side effects of vaccine and erroneous ideas of vaccine contraindications were some of possible the reasons for incomplete immunization. Vaccine hesitancy is defined as delay in acceptance or refusal of vaccine despite the availability of vaccination service, and is one the reason for incomplete immunization[41].Other studies from Ethiopia support this and mothers hesitate to vaccinate their children because of fear of side effects, excessive waiting time, hearing rumors about vaccination and fear of the vaccine needle[42, 43]. Vaccine hesitancy studies from Italy revealed that the main reasons for vaccine hesitancy was not trusting the information given about vaccines and that not vaccines are important [44, 45].

Children of older mothers were more likely to be fully immunized. As the maternal age increases the child immunization coverage also improved. This may because older mothers may have experience and better knowledge of the effect and importance of immunization compared to younger. This is also in agreement with studies from Ethiopia [25, 46] and other African countries [18, 47–49].

Our findings also show that use of antenatal care was associated with full immunization coverage, as mothers with one or more tetanus toxoid vaccines during pregnancy were more likely to be fully immunized. These results correspond with those documented elsewhere in Ethiopia [20, 21, 36, 50, 51] and Africa [37, 52–54]. Follow-up care at a health institution during pregnancy and having one or more tetanus toxoid vaccine creates opportunities to obtain adequate information to discuss vaccines and vaccine-preventable diseases and to encourage adherence to vaccine schedules [55–57].

Moreover, mothers who knew the age at which a child should complete immunizations tended to have children with high immunization coverage, as has been documented from Ethiopia [21, 22, 27] and Nigeria[49]. This may explain that mothers who know age at which the child should complete immunization is likely to be fully immunized and may get information from health facility or through media or other sources. We also found that girls had higher immunization coverage than boys, which is in line with findings of studies in Ethiopia and Ghana [5, 18, 53]. In other study, boys were more likely to be fully immunized[22]. However, the reason why there is variation in coverage with gender is not evident.

Community-level factors associated with vaccination were highlighted in this study. We observed significant difference in immunization coverage across communities. Specifically, children from the same kebele tended to have similar immunization status. Our study also showed that vitamin A supplementation was associated with higher vaccination status, as others have found [36]. Vitamin A campaigns in the community may increase awareness about vaccinations. Strengthening the outreach and campaign program will improve the immunization coverage.

The study has some limitations. The survey was cross-sectional, so causal relationships between variables of interest could not be assessed with certainty. Because the data were self-reported by the mothers, it is possible that responses were affected by recall and social desirability bias. The survey study may also have anon-response error. The study has failure to assess the degree to which non-response error is likely to have.

As study strength, the study is population based and used multilevel multivariate regression analysis.

## Conclusions

There is low immunization coverage in the Wonago district of southern Ethiopia. We found that individual and community factors affect the childhood immunization. Our study suggests the need for policies that address low immunization coverage. These awareness programs should emphasize the importance of health care access and use for mothers and children and of immunization schedules.

## Supporting information

S1 File Questionnaire used to conduct assess immunization coverage data collection in Wonago district in southern Ethiopia, June 2017- July 2017. (ZIP)
