# Supplementary material for: Low immunization coverage in Wonago district, southern Ethiopia: A community-based cross-sectional study
Source: PLoS One. 2019 Jul 24;14(7):e0220144. doi: 10.1371/journal.pone.0220144 (PMC6655723; doi:10.1371/journal.pone.0220144)
Supplement: S1 File — (ZIP) [file pone.0220144.s001.zip › Questionnaire English version.pdf]

Hello. My name is ______________________________________ and I am working with the with the research entitle" Assessment of common childhood illness, immunization coverage and health service utilization of under five children" . You are selected to participate in this survey. We would very much appreciate your participation in this survey. This information will help the government to plan health services. First we will ask some questions about your child and household. The survey usually takes between 30 and 40 minutes to complete. And follow up survey for the next 6 months that will takes 10-15 minutes. During the survey there will be assessment and measurements and some of the questions that you need to see and report for us also.

Whatever information you provide will be kept strictly confidential, and will not be shared with anyone other than members of our survey team.

Participation in this survey is voluntary, and if we should come to any question you don't want to answer, just let me know andI will go on to the next question; or you can stop the interview at any time. However, we hope you will participate in the surveysince

The files will be kept confidential in the file cabinet of the project office for five years. Only the researchers will use files. If there is any explanation about any issues you can get the principal investigator with mobile number of 0911910959.

Your views are important.

At this time, do you want to ask me anything about the survey?

Do you agree to participate in the study?   Agree ……..1   Disagree……..2

Participant signature/left finger --------------------------

Thank you for your participation!

Code------------------

Zone	----------------------------
Name of district	-----------------------
Kebele	1.Hasse Harro 2. Mokonisa 3. Tumattaa cherecha
Locality name/Gote	--------------
Cluster Number/1 to 5	
Name of Household Head	
Household Number/House ID	
Date and Time of interview started	Date-----------Time------------minute----
Date and Time of interview finished	Date-----------Time------------minute----
Interview name and siginture	---------------------- ---------------------------------
Supervisor Name and signiture	---------------------- ---------------------------------
Office editor name and signiture	---------------------- ---------------------------------
Result of quetioner	1 2 3 4 5 6 7
Distance from locality to health organization	Health post---------- km Healthcenter---------km Hospital--------km
Time that take from locality to health organization	Health post---------- min Healthcenter-------min Hospital--------min

Result questionnaire

1. Completed
2. No household member at home or no competent respondent
3. Entire household absent for extended period of time
4. Postponed
5. Refused
6. Dwelling vacant or address not dwelling

Code----------------------------

Part one ; Mother's or care taker's background information		
	Code of the mother	---------------------
101	How old are you?	---------------years
102	Are you able to read or write a simple sentence?	Yes……….1<br>No……….2
103	Did you ever attend formal school?	Yes……….1<br>No……….2
**If question number 103 answer is no skip to question number 105**		
104	what is the highest class level	Primary (1-8)................1<br>Secondary(9-10)..............2<br>Preparatory(11-12)...........3<br>College/university.................4<br>Other specify...........9
105	What is your current marital status?	Single ……........1<br>Married ....2<br>Divorced/Separated............. 3<br>Widowed................... 4
106	What is your religion?	Orthodox . . . . 1<br>Protestant . . . . 2<br>Catholic . . . . 3<br>Muslim . . . . 4<br>Traditional . . . . 5<br>Specify other--------------6
107	What is you ethinc group	Gedeo.............1<br>Oromo ....2<br>Sidmma...........3<br>Woleyeta..............4<br>Other specify 5
108	What is you occupation	Housewife...............1<br>Private worker ....2<br>Student.......................3<br>Merchant...................4<br>Daily labour.................5<br>Farmer.......... 6<br>Government employed............ 7
109	Did your husband ever attend school?	Yes……….1<br>No……….2
**If question number 109 answer is no skip to question number 111**		
110	what is the highest class level of	Primary (1-8)................1

	your husband	Secondary(9-10)................2<br>Preparatory(11-12)...........3<br>College/university.................4<br>Other specify...........5
111	What is your husband occupation?	Farmer………..…01<br>Farming other land……… 02<br>Merchant……… ……… 03<br>Dailylabour…… 04<br>Government employed…. .05<br>Professional……… 06<br>In army............... 07<br>Retired…….. 08<br>Other specify 09
112	Number of mother delivered/parity	--------------------in number
113	Total number of household member	--------------------in number
114	Total number of under five children in the house	------------------in number
115	Death of under five children in the past one year	-----------------in number
116	What is the birth interval between the childrens?	---------------------years
117	Do you use any type of contraceptive? (Now)	Yes……….1<br>No……….2
**If question number 117 answer is no skip to question number 119**		
118	What type of family contraceptive do you use?	Pills............1<br>Condom.........2<br>Injection.........3<br>Implant...........4<br>IUCD...........5
119	Who decied in caring of child in the house?	Husband………..1<br>Wife…..……2<br>Both husband and wife………..3<br>Other specify.............4
120	Do the Health extension worker visit you with her home to home visit?	Yes……….1<br>No……….2
121	Is the household participated in the safety net program	Yes……….1<br>No……….2

Code---------------------

Part two: List of under five children

	List of under five child in the household	Nam	Name	Name
201	Child code	-------------------	--------------------	---------------
202	Age in month	In month---------	In month---------	In month---------
203	Sex of Child<br>1. Boy 2. Girl	1 2	1 2	1 2
204	Birth order (birth sibling) of the child ?<br>First……..1<br>Second ….2<br>Third……3<br>Fourth…….4<br>Fifth ……5<br>Sixth and above…….6	1 2 3 4 5 6	1 2 3 4 5 6	1 2 3 4 5 6

**Part 3 Prenatal, delivery services and newborn care**				
		Name	Name	Name
	**Child code**			
301	When you were pregnant with [NAME], did you go to a health facility for antenatal care?<br>Yes….1 No…...2	1 2	1 2	1 2
**If question number 301 is no ,skip to question number 304**				
302	How many times did you receive antenatal care during your pregnancy with<br>Number________<br>Do not remember.......................98	------------	------------	------------

303	Where did you receive antenatal care for this pregnancy?<br>Home...……..1<br>Government hospital…..……..2<br>Health cener...............3<br>Health post................4<br>NGO clinic...............5<br>Private clinic…..……..6<br>other private organization..………7<br>Other specify……..…8	1 2 3 4 5 6 7 8----	1 2 3 4 5 6 7 8-------	1 2 3 4 5 6 7 8---------
304	When you were pregnant with (Name), did you receive any injection to prevent him or her from getting tetanus that is convulsions after birth (an anti-tetanus shot, an injection at the top of the arm or shoulder)?<br>Yes….1 No..…...2	1 2	1 2	1 2
305	Did you see anyone for antenatal care of pregnancy?<br>Yes….1 No..…...2	1 2	1 2	1 2
**If question number 305 answer is no skip question number 307**				
306	Whom do you see/care you for ANC visits?<br>Health professionals.............1<br>Trained birth attendants …..……2<br>Untrained birth attendants ………3<br>Voluntary Community Health Worker (VCHW) ………4<br>Other specify ………5	1 2 3 4 ----------5	1 2 3 4 --------------5	1 2 3 4 --------------5
307	Where did you give birth to (Name)?<br>Home…………..1<br>Government hospital…………..2<br>Government health center…………..3<br>Health post…………4<br>NGO clinic…………5<br>Private clinic …………6<br>Other specify………..7	1 2 3 4 5 6 ----------------7	1 2 3 4 5 6 ----------------7	1 2 3 4 5 6 ----------------7
308	Who assisted with the delivery of (name)?<br>Health professionals...............1<br>Trained birth attendants..………2<br>Untrained birth attendants ……..…3<br>Relative/Friend/Neighbor…..…….4<br>No One…………5<br>Other specify…………6	1 2 3 4 5 6------------	1 2 3 4 5 6---------------	1 2 3 4 5 6-----------------

309	Is Health extension worker/health professional visist you in the frist two week,after delivery<br>Yes………1 No………..2	1 2	1 2	1 2
310	Have you exclusively breast feed in the frist 6month<br>Yes……………1 No……….2<br>3. Sill breast feed	1 2 3	1 2 3	1 2 3

Code-----------------------

Part 4. Child health and nutrition status				
		Name of child	Name of child	Name of child
	Child code			
401	Has [Name] been ill at any time in the last 2 weeks?<br>Yes…….1 No……..2	1 2	1 2	1 2
**If question number 401 answer is no skip to question number 425**				
402	Is yes,what was the illness of the child<br>Diahrrea………1<br>Cough..… …. 2<br>Fever……......3<br>Other (Specify) 4	1 2 3<br>4------------	1 2 3<br>4--------------	1 2 3<br>4--------------
403	Has (Name) had diarrhea/watery diarrhea stays more than 3 days/ in the last 2 weeks?<br>Yes……1 No.……..2	1 2	1 2	1 2
**If question number 403 answer is No skip to question number 414**				
404	For how long the diarrhea last?<br>--------------------------Days	--------------------<br>Days	------------------<br>Days	-------------------------<br>Days
405	Was there blood in the stool?<br>Yes…….1 No.……..2	1 2	1 2	1 2
406	If the child had diarrhea: was [Name] given any of the following to drink?<br>ORS packet ………1<br>Home made sugar and salt solution ……... 2<br>Other homemade fluid..……3<br>No fluid drink....................4	1 2 3 4	1 2 3 4	1 2 3 4
407	Did you seek advice or treatment for the diarrhea from any source?<br>Yes…….1 No.……..2	1 2	1 2	1 2

**If question number 407 answer is No skip to question number 410**				
408	Where did you seek advice or treatment?<br>Government hospital……..1<br>Government health center………2<br>Healthpost ..…….3<br>NGO clinic.............4<br>Private clinic.………5<br>Other private…………6<br>Drug vendor/store………..7<br>Shop.………8<br>Traditional healers………9	1 2 3 4 5 6 7 8 9	1 2 3 4 5 6 7 8 9	1 2 3 4 5 6 7 8 9
409	Within what time (name), you take to the place where you seek care?	Day----------	Day----------	Day----------
410	What (else) was given to treat the diarrhea?<br>Antibiotics…………. 1<br>Ant motility……………….2<br>Zinc……………….. 3<br>Home Remedy Rice Starch ....4<br>Nothing is given....................5<br>Other specify…………..6	1 2 3 4 5<br>6-----------	1 2 3 4 5<br>6-------------	1 2 3 4 5<br>6-----------
414	Has (Name) been ill with fever at any time in the last two weeks?<br>Yes..…….1 No.……..2	1 2	1 2	1 2
415	Did [Name] sleep under a bed net last night?<br>Yes…….1 No.……..2	1 2	1 2	1 2
416	Has (Name) had an illness .with cough at any time in the last 2 weeks?<br>Yes…….1 No.……..2	1 2	1 2	1 2
**If question number 416 answer is No skip to question number 419**				
417	How many days did cough lasts?	----------------days	----------------days	----------days
418	When (Name) had an illness with a cough, did she/he breath faster than usual with short breaths or have difficulty breathing?<br>Yes…….1 No.……..2	1 2	1 2	1 2
419	Do child(Name) has fast breathing See the breathing and count in one minute	________<br>Breath in one minute	________<br>Breath in one minute	________<br>Breath in one minute

420	Which part of the respiratory organ do the Child(Name) had difficulty breathing<br>Chest only………1<br>Nose only……….2<br>Both nose and chest……3<br>I do not know …..4	1 2 3 4	1 2 3 4	1 2 3 4
421	Do the child (Name) has runny nose?<br>Yes ….1 No…2 I do not know…3	1 2 3	1 2 3	1 2 3
422	Did you seek advice or treatment for the illness from any source?<br>Yes…….1 No……..2	1 2	1 2	1 2
**If question number 422 answer is No skip question number 426**				
423	Where did you seek advice or treatment?<br>Government hospital……..1<br>Government health center….2<br>Health post…….3<br>NGO clinic.............4<br>Private clinic………5<br>Other private…………6<br>Drug vendor/store………..7<br>Shop…………8<br>Traditional healers……….9	1 2 3 4 5 6 7 8 9	1 2 3 4 5 6 7 8 9	1 2 3 4 5 6 7 8 9
424	Within what time (name), you take to the place where you seek care?	Day----------	Day----------	Day----------
425	What type of medication do the child (Name) had taken?<br>Anti-malaria……..1<br>Antibiotic………..2<br>Anti-pain…………3<br>Other medicine……4	1 2 3 4		
426	Did you cost on the out-patient /inpatient/traditional treatment of the illness for your child?<br>Yes……..1 No…………2	1 2	1 2	1 2
**If question number 426 is No skip to question number 428**				

427	cost on the out-patient /inpatient/traditional treatment of the illness for your child<br>**(Local price)**<br>1. Card<br>2.Drug<br>3.Laboratory<br>4.Consultation<br>5. Transport cost to and from health facility<br>6. Additional expense for care giver<br>7.Hospitilization<br>8. Traditional healer<br>9.Total cost	Cost in birr<br>1.-------------------<br>2.-------------------<br>3.-------------------<br>4.-------------------<br>5.-------------------<br>6.-------------------<br>7.------------------<br>8.-------------------<br>9.----------------	Cost in birr<br>1.-------------------<br>2.-------------------<br>3.--------------------<br>4.--------------------<br>5.--------------------<br>6.--------------------<br>7.------------------<br>8.---------------------<br>9. --------------------	Cost in birr<br>1.-------------------<br>2.-------------------<br>3.--------------------<br>4.--------------------<br>5.--------------------<br>6.--------------------<br>7.------------------<br>8.---------------------<br>9-----------
428	If the child did not seek advice or treatment , what was the reason?<br>Percived resolved by itself.............1<br>Lack of money................2<br>Illness was not sever enough ....3<br>Did not trust by treatment...............4<br>Distance of the facility................5<br>Other specify....................6	1 2 3 4 5<br>6-------------	1 2 3 4 5<br>6---------------	1 2 3 4 5<br>6-------------------
429	In the past 6 month of National immunization date do the child take Vitamin A?<br>Yes........1 No…....2	1 2	1 2	1 2
430	In the past 6 month of National immunization date do the child take deworming? Age more than 2 year<br>Yes….......1 No......2	1 2	1 2	1 2
				Code ------------------------

**Anthropmetry measurment fo children age under five children**				
		Name of child	Name of child	Name of child

	Child code			
431	Weight in Kg	________________<br>Kilo gram	________________<br>Kilo gram	________________<br>Kilo gram
432	Height in CM	-----------------<br>Centi meter	-----------------<br>Centimeter	-----------------<br>Centi meter
433	MUAC of Child<br>Age greater than 6 month	---------------<br>Centimeter	---------------<br>Centimeter	---------------<br>Centimeter

Code--------------------

**Part 5 Immunization status of child in age group 6-36 month**				
		Name of the child	Name of the child	Name of the child
	Child code			
	Age in month	----------------- month	------------------ month	------------ month
501a	Ever heared about immunization<br>Yes……..1 No…....2	1 2	1 2	1 2
501b	From where you heared the information?<br>Health professional…..........1<br>Friends............2<br>Radio….........3<br>Televsion….......4<br>School….........5<br>Neighbour..........6	1 2 3 4 5 6	1 2 3 4 5 6	1 2 3 4 5 6
501c	When did the chid start immunization?<br>At birth.............1<br>After one month.. ....2<br>I do not know............3	1 2 3	1 2 3	1 2 3
501d	To complete immunization how many times should child immunize?<br>One time…..........1<br>Two times...........2<br>Three times............3<br>Four times...........4<br>Five times ....5<br>I do not know........6	1 2 3 4 5 6	1 2 3 4 5 6	1 2 3 4 5 6
501e	Age to complete immunization<br>Before one year.............1<br>One year and more…..........2<br>I do not know…........3	1 2 3	1 2 3	1 2 3
**502**	**Attitude of the mother to immunization**			
502a	Immunization program is free<br>Agree……....1<br>Disagree…........2	1 2	1 2	1 2
502b	Immunization prevent disease<br>Agree……....1			

	Disagree….........2	1 2	1 2	1 2
502c	Immunination is necessary for children<br>Agree…........1<br>Disagree….......2	1 2	1 2	1 2
502d	Immunization is given for easy disease<br>Agree……....1<br>Disagree...........2	1 2	1 2	1 2
502e	Sideeffect of immunization is very serious<br>Agree……....1<br>Disagree….........2	1 2	1 2	1 2
502f	Immunization make the child to be ill<br>Agree……....1<br>Disagree……....2	1 2	1 2	1 2
503	Did the child ever immunizaed?<br>Yes……….1 No……...2	1 2	1 2	1 2
**If question number 503 answer is No skip to question number 516**				
504	Did you ever have vaccination card for the child<br>Yes……….1 No……...2	1 2	1 2	1 2
**If question number 504 answer is No skip to question number 507**				
505	Does the child have a scar from BCG vaccination<br>Check for BCG scar<br>Yes……….1 No……...2	1 2	1 2	1 2

<table>
<tr><td>506</td><td>Copy vaccination date for each vaccine from the card

Write “44” in “Year” column if card shows that a vaccination was given, but no date is recorded</td><td>Date Month Year
BCG ___|______|___
Polio 0___|______|__
Polio 1___|______|__
Polio 2___|______|__
Polio 3____|_____|__
DPT1___|______|___
DPT2___|______|___
DPT3___|______|___
PCV1___|______|___
PCV2___|______|___
PCV3____|______|__
Rota1___|______|___
Rota 2___|______|___
Measle ___|____|___
Vitamin A/___|____|_</td><td>Date Month Year
BCG___|______|___
Polio 0___|______|_
Polio 1___|_____|_
Polio 2___|____|_
Polio 3____|____|__
DPT1___|______|__
DPT2___|______|__
DPT3___|______|__
PCV1___|______|__
PCV2___|______|__
PCV3____|____|___
Rota 1____|____|_
Rota 2__|_____|____
Measle ___|____|___
Vitamin A/___|___|_</td><td>Date Month Year
BCG___|_____|____
Polio 0___|______|_
Polio1___|______|_
Polio 2___|____|___
Polio3____|____|___
DPT1___|______|__
DPT2___|______|__
DPT3___|______|__
PCV1___|______|__
PCV2___|______|__
PCV3____|_______|_
Rota 1 ___|____|___
Rota 2 ____|___|___
Measle ___|____|___
Vitamin A___|____|_</td></tr>
<tr><td colspan="5">Woman has no vaccination card of the child</td></tr>
<tr><td>507</td><td>A BCG vaccination against tuberculosis that is an injection in the arm or shoulders that usually causes a scar?
Yes..........1 No.......2</td><td>1 2</td><td>1 2</td><td>1 2</td></tr>
<tr><td>508</td><td>Check for BCG scar.is thee?
Yes..........1 No.......2</td><td>1 2</td><td>1 2</td><td>1 2</td></tr>
<tr><td>509</td><td>Polio vaccine, that is, drops in the mouth, do the child take polio vaccine?
Yes..........1 No.......2</td><td>1 2</td><td>1 2</td><td>1 2</td></tr>
<tr><td colspan="5">If question number 509 answer is no skip to question number 512</td></tr>
<tr><td>510</td><td>When was the first polio vaccine received, just after birth (within 2 weeks) or later?
Just after birth……………1
Later than two weeks..…………...2</td><td>1 2</td><td>1 2</td><td></td></tr>
</table>

511	How many times was the polio vaccine received? Number_______<br>Do not know...............98	_____98	_______98	_______98
512	DPT vaccination, that is, an injection given in the thigh or buttocks, sometimes at the same time as polio drops. Do the child take DPT vaccine<br>Yes…………1 No…………...2	1 2	1 2	1 2
**If question number 512 answer is No skip to question number 514**				
513	How many times was the DPT vaccine received?<br>Number_______	_______	_______	_______
514	PCV vaccination given in the thigh pr buttock to prevent pnemona<br>Yes…....1 No…...2	1 2	1 2	1 2
515	Rota vaccine which is used for prevent dihrrea, arough drop<br>Yes…….1 No…...2	1 2	1 2	1 2
514	An injection to prevent measles, given around 9 months of age?<br>Yes…….1 No…....2	1 2	1 2	1 2
515	Since your child is fully immunized, did (Name) receive the immunization diploma?(*Show Immunization Diploma)*<br>Yes…….1 No…....2	1 2	1 2	1 2
516	**Look back at the information on the child's immunization card or the information given by the mother**<br>What is child immunization status?	1 Fully immunzed<br>2. Not immunize<br>3 . Not fully immunized<br>4. Completed for his/her age	1 Fully immunzed<br>2. Not immunize<br>3 . Not fully immunized<br>4. Completed for his/her age	1 Fully immunzed<br>2. Not immunize<br>3 . Not fully immunized<br>4. Completed for his/her age
517	If the child not immunized or partially immunized, what was the reasons?			

	Unaware of need immunization……1<br>Unaware of need to return for 2nd or 3rd dose......... 2<br>Place and/or time of immunization Unknown ....3<br>Fear of side reactions……….. 4<br>Wrong ideas about contra-indications……....5<br>No faith in immunization………6<br>Place of immunization too far……..7<br>Time of immunization inconvenient…8<br>Vaccinators is absent.………..9<br>Vaccine not available…………...10<br>Child ill and not brought………11<br>Long Waiting time…..…….12	1 2 3 4 5 6 7 8 9 10 11 12	1 2 3 4 5 6 7 8 9 10 11 12	1 2 3 4 5 6 7 8 9 10 11 12
