# Supplementary material for: Low immunization coverage in Wonago district, southern Ethiopia: A community-based cross-sectional study
Source: PLoS One. 2019 Jul 24;14(7):e0220144. doi: 10.1371/journal.pone.0220144 (PMC6655723; doi:10.1371/journal.pone.0220144)
Supplement: S1 File — (ZIP) [file pone.0220144.s001.zip › Questionnaire Gedeoffa version.pdf]

Ifisanjonna eeti qoratixxa qiste

Ulfesendeeki Aaddi/Baabba

Nage'inginnigaldine?! Ani --------------------------------------hiyyemannon. Onde wogganni butti'a hexxeexxi oose elagophaxxe dhibonaa'n qufa'a,eebbinjo, albaatenna sagaletixxe heeno'manni) ;kittibatet jeejinna fayyatixxa la'o uwwa hana assendaaxxe towacho'a tarja bukki assaten፡ Ati tenne towacho'n afematte'e mellendeteen.Tenne towe giddo afendattoki kinni atixxi eet hexxaa wodda callan. Edisaaxxi belt/o yookin anno atixxanna mini hadixxa una iittanni 6 agenjo giddo anno hordoffe alfitee qico qortumik lame-lame torba'n qorraannen. Taakkaka tarja bukkasate'e 40 daqiiqa iittanni kinni hordofetixxi yanna yo'oxxa yanna 10-15 daqiiqi qico'n turtaan.Hordofetixxa yanna qortumikinna mika hexxaan. Atenaa'n heqqendaaxxi qorraaka qortuma hiissa; annotika qufa'a; albaate, eebbinjo, hooneenna bisoke'n jijjiirra hexxeexxa borreessat weldeennatixxe hundaayye'n afema hasissaan.

.Ate'n aagebaaxxanna gibiseeka hoone qortumanna hiisa gopha dandetatton.Iittanni kinni konne qortuma fetetexxe yanna'n uurisa dandetatton.Towachot giddo ati afema gophiyyo heeneenna fayyatenaa'n alfitatoxxa la'o ate'e gophisabaan. Yaane kinni qortumake'e hiissitatoxxi dhugati hiissichchi rakko egenannoshsha xuullo hasate'e, herregatenna hujete'n hosisate'e gargaraan.Ati uutatokki tarji qo'nete'e male wele huje'a hosaakebaan.

Tenne qo'nexxe kuta kadat hiinina rakko geya dandetabaan. Qo'ne tenne'n afema kinni ati'a la'o affaan hinee addantannon.Yaane kinni qo'net tarji annotixxa fayya heqqate'e gargaraan.Qortumake'e hasisaaka hiissiichcho uutineexxe yo'osha ass'ne'e galatenafantaannen. Ifisate'e hassaateki qortumi hedhoole qorte qo'nisallo Samirawit Hayiluxxa konne siliketike bilibilinni (0911910959) qora dandetatton. Tenne towacho'n afemate'e eetina'ne?

Eete……………..1 Waawwot……………..2

Qoraakika beesisa ………………………..

Qoremaakika beesisa …………………

Codee------------------------

Zonne --------------	
Rogii ----------------	
Kebeleee	1.Hasse Harro 2. Mokonissa 3. Tumattaa cherrecha
Looolink summi	----------
Gollot/1;5^{xx}i laakkoossi	----------
Miniinik anni summi	----------
Minii laakkoossi	
Qortumi kunni assemeexxi	Barra------------yanna -------------Dquqa----
Qortumi uurrexxi	Barra------------yanna -------------Dquqa---------
Annichchi qortetik	
Hordofalli	
Biirotik hordofalli	
Qortetixxi alfa	1 2 3 4 5 6 7
Cilotik mini hittee qico bayyata?	Xeena keela------------KM Xeena xaaba----------KM Hoosipitaala --------KM
Cilotik mini hittee qico yanna adahann?	Xeena keela----------- Dquqa Xeena xaaba---------- Dquqa Hoosipitaala -------- Dquqa

Qortetixxi alfa

1. Muuxxendeexxi
2. Hiissichcho uwwaaki hadi gophema
3. Mini hadi duuchchixxe yanna'n gophema
4. Wele yanna'a weldeenna abida
5. Mini hadi hissa gibben
6. Mini gophema

Codee-----------------------

Hafuura adhdha 1; Amatikanna oose badadaake duuchcha tarja		
	Ammatixxi codde	-----------------------
101	Wogga atixxi me'ete?	-------------- Wogga
102	Yaansisa/nabbaba yookin borreess dandeetetatto?	Eet……….1<br>Wawwot……….2
103	Deettitte barachcho hordoffe egendete?	Eet… …….1<br>Waawwot…….2
**Qortuma 103 waawwot kadoole qortuma.105 sa'we**		
104	Hordofineexxi ege'dine? kaddoole muuxxinxi kuta me'ete?	1^xxa^coobba (1-8)................1<br>2^xxa^ coobba(9-10)................2<br>Preparratory(11-12)…............3<br>Colleegye/universiitee...............4<br>Wele hexxoole kulle.............9
105	Ta'a hexxineeki bultetiki jeeji hitta hedheeke?	Heerundebaaxxe……… 1<br>Heerundeexxe……….. 2<br>Heerundeexxi tikendeexxi................3<br>Miniki anni reyeexxi............ 4
106	Heene adde hordofatto?	Ortodokisse……….. 1<br>Pirotesintaane………. 2<br>Kaatoolike ………..3<br>Islaama………..4<br>Aadatixxa……….5<br>wele hexxoole kulle............6
107	Heene adde gossaa**?**	Gedeeoxxi.............1<br>Oromooxxi ....2<br>Sidmaaxi ....3<br>Woleyetaa..............4<br>wele hexxoole kulle 5
108	Ati maachcho huja'ne?	Minnixxi amma.............1<br>Issixxa huje affek ....2<br>Barrato.......................3<br>Daddalto ....4<br>barratike hujallo ....5<br>Wiisallo .... 6<br>Mootumatixxa huje'a 7
109	Mini anni atiki barachcho hordofeexxi ege'ne?	Eet……….1<br>Wawwo……….2

**Qortuma 109 waawwot kadoole qortuma.111 sa'we**		
110	Barachcho hordonfeexxi egeneexxe kadoole፣<br>Lumoke koobbixxa barachcho muuxxeeki me'et?	1^{xxa}coobba(1-8)................1<br>2^{xxa} coobba(9-10)................2<br>Preparratory(11-12)...........3<br>Colleegye/universiitee.................4<br>wele hexxoole kulle 5
111	Miniik anni ati maachcho huja'ne?	Wiisallo…………..01<br>Wele mannika fichcha huja……… 02<br>Daddalto… ……… 03<br>barratike hujallo…… 04<br>Mootumatixxa huje'a…. .05<br>Ogeessa……… 06<br>Sannachcha bogiinke……. 07<br>Qulula…….. 08<br>Wele hexxoole ifisse 09
112	Duuchchi meeye oose elidexxe?	--------------------Laakkoossik
113	Duuchchi mini giddo hedhaaki mini hadixxi heddunte laakkoossik	--------------------Laakkoossik
114	Duuchchinxxi 5 woggan butti'a hexxeexxi oose heddunte laakkoosik	------------------Laakkoossik
115	5 woggan butti'a hexxeexxi reeaxxi oose	-----------------Laakkoossik
116	Oosetixxe oddo'n hexxeexxi elenshatixxi gargarunte?	--------------------wogga
117	Mini hado minkeexxi ilatixxa barachcho la'effatte?	Eet..................1<br>Waawwo.................2
**Qortuma 117 waawwot kadoole qortuma.119 sa'we**		
118	Etta fekaataxxi eilla qerrishach /urrussach adhitaaeat?	Kiniine..................1<br>Kondoome............2<br>Liimmo/marfe.........3<br>Angatixxe ciqile'n...........4<br>Luuppe/gadameesi giddo...........5
119	Anno unusate'e mini giddy oose unuunsinaashsha assaaki aate?	Anni/aro'i/…………..1<br>Aree…………2<br>Arena aro'i………..3<br>Wele hexxoole ifisse/...........4
120	Xeenna extentionne minne minnenn uushoo dagggaa ?	Eet.................1<br>Waawwo.................2
121	Mini hado safeteey netixa pogrammee gido heren?	Eet..................1<br>Waawwo.................2

Codee-------------------------

Hafuura adhdha leme ; Wogga 5 butti'a kaddeexxi

	Wogga 5 butti'a kaddeexxi	Summa	Summa	Summa
201	Annotixxi Codee	--------------------	---------------------	----------------
202	Wogga agenjo	-------- Agenjo	-------- Agenjo	-------- Agenjo
203	Kulemaakika saala<br>1.Labalo 2. Melale	1 2	1 2	1 2
204	Mini gidoEelenshatee meekaxxi? Mekkaba elende<br>Angaffa.............1<br>Langaaxxi............2<br>Sakaxxee..................3<br>Sholegatxxe............4<br>Onndaxxe.........5<br>JannexxenaWell heredhole ifissi…….6	1 2 3 4 5 6	1 2 3 4 5 6	1 2 3 4 5 6

**Hafuura adhdha 3; siili edidarrexxa ,ilatixxenna laattummatixxa la'o uuddaaxxa**				
		Annoti summi	Annoti summi	Annoti summi
	Annotixxi Codee			
301	Silii wodda hordoffe cilotike/hakimetie mine'n assitaata?<br>Eet…….1 Waawwot……..2	1 2	1 2	1 2
**Qortuma 301 waawwot kadoole qortuma.304 sa'we**				
302	Duuchcha yanna hordoffe assiteteexxi hiteexxe?<br>Laakkoossik ________<br>Kasseyehabonano...................98	------------	------------	------------

303	Silii woddixxa hordoffe assiteteki habate?<br>Mini giddo………..1<br>Hoosipitaala ardinke'n…........2<br>Xeena xaaba mootumatike'n….......…3<br>Xeena keela……….4<br>Mootumatixxe kadebaaxxe orga<br>Kilinike…........5<br>annichchinke / kilinike………6<br>wele annichchinxe orga……..…7<br>wele hexxoolenna kulle..………8	1 2 3 4<br>5 6 7 8----<br>----	1 2 3 4 5<br>6 7 8--------	1 2 3 4 5 6<br>7 8---------
304	Silii wodda mengaagga qolfixxa kittibaate adhdhitetexxi yanna hexxe?(angate'n uunaaxxa mengaagga qolfixxa kittibaate<br>Eet……1 Waawwot ..…...2	1 2	1 2	1 2
305	Siilinxxe hordofetixxe yanna'n ate'e hordofaaki aatemma**?**<br>Eet……..1 Waawwot ……..2	1 2	1 2	1 2
**Qortuma 305 waawwot kadoolee qortuma akkoossa.307 sa'we**				
306	Siilinxxe yanna'n ate'e badadeeki aate?<br>Fayyatixxe ogeeyye.....................1<br>Leenjisendeexxi awaaleje…….……2<br>Leenjisendebaaxxi awaalaaje……….3<br>Aradiinki toliika kipha assaa............…4<br>Wele hexxoole kulle………..5	1 2 3 4<br>-----------5	1 2 3 4<br>--------------5	1 2 3 4<br>--------------5
307	Anno haba ileme?<br>Mini giddo …………..1<br>Hosipitaala…………..2<br>xeena xaaba...............…3<br>xeena keella……………4<br>Mootummatixxe kaddebaaxxe Orga<br>kilinike………5<br>Annichchinka Kilinike …..6<br>Wele nna hexxoole ifisse……..7	1 2 3 4 5<br>6<br>----------------<br>7	1 2 3 4 5<br>6<br>----------------<br>7	1 2 3 4 5 6<br>----------------<br>7
308	Annotika ilatixxa yanna kiphaaki aate?<br>Fayyatixxi ogeeyye...……….1<br>leenjinedeexxi awwaalaaje……........2<br>Leenjisendebaaxxi awaalaaje……..... 3<br>firi/jaali/olla'i……….4<br>ayyinna…………5<br>welinna hedhoole iffisse……….6	1 2 3 4 5<br>6------------	1 2 3 4 5<br>6--------------	1 2 3 4 5<br>6----------------
309	Ildetexxe taakkaaxxe torba'n xeena ekisitenshiine dageexxi uudde?<br>Eet…………1 Waawwo………...2	1 2	1 2	1 2
310	Anno meaye agenjjon unnuna ewetete?<br>Eet…………1 Waawwo………..2<br>Tanaa'nna unnunssean heren……..3	1 2 3	1 2 3	1 2 3

Codee ---------------------

Hafuura 4; Oosetixxa fayyatikanna sagaletika jeeja				
		Annoti summi	Annoti summi	Annoti summi
	Annotixxi Codee			
401	**Anno saxxeexxe 2 torba giddo ela gophee egende?**<br>Eet…….1 Wawwo……..2	1 2	1 2	1 2
**Qortuma 401 waawwot heetolee qortuma.425 sa'we**				
402	Anno ela gopheexxe kaddoole ise'e dhibeechchi maachcho?kulle<br>Albaate..................1<br>Qufa'a…………… 2<br>Eebbinjo …........……3<br>wele hexxoole ifisse…….……….4	1 2 3<br>4------------	1 2 3<br>4------------	1 2 3<br>4--------------
403	Anno saxxeexxa lemme torba yanna albaatemma?<br>Eet…………..1<br>Waawwot…………..2	1 2	1 2	1 2
**Qortuma 403 waawwot kadoole qortuma .414 sa'we**				
404	(Anno summi) albaata meeyye barra<br>-------------------------Barra	--------------------	------------------	---------------------
405	Ciloke'n mude makendeemma?<br>Eet………1 Waawwot……2	1 2	1 2	1 2
406	Anno albaateexxe kaddoole maachcho ha'witaachcho uutine? kulle<br>ORS ………1<br>Sukkaaranna maxinemakat..….. 2<br>weleba giisemeeka wode'e……3<br>Weelee giisemeeke wode'e addeheban............4	1 2 3 4	1 2 3 4	1 2 3 4
407	Albaatexxa dukuba fayuuntaxxi mine meeyea?<br>Eet……..1 Waawwot……….2	1 2	1 2	1 2
**Qortuma 407 waawwot kadoole qortuma .410 sa'we**				

408	Hoone cilotike mine'n Ci'lo habaa'n alfitette?<br>Hoosipitaala ardinke'n..........1<br>Xeena xaaba mootumatike'n…....…2<br>Xeena keela…..….3<br>Mootumatixxe kadebaaxxe orga Kilinike…........4<br>Annichchinke / kilinike ..…….5<br>wele annichchinxe orga……….....6<br>Suqee…………7<br>Qorsii mini…..…….8<br>Aadatixxa ci'lo……..….9	1 2 3 4 5 6 7 8 9	1 2 3 4 5 6 7 8 9	1 2 3 4 5 6 7 8 9
409	Anno alebaateten hitteexxa yanna'n cilotika mine geesisete?<br>--------------------------Barra	----------------------<br>Barra	-----------------------<br>Barra	______________________<br>Barra
410	Anno albaate wodda wele qorsa uutineeki hedhe?<br>Bacetrixxa qorsa…..…. 1<br>Ant motility……….2<br>Zinkee……….. 3<br>Mini giddo qixxesemeeka ruuzetika wode'e............4<br>Anno mitichina addehban…......5<br>Weli hedhoole ifise.....................6	1 2 3 4 5<br>6-----------	1 2 3 4 5<br>6-------------	1 2 3 4 5<br>6-----------
414	Saxxeexxe lame torba giddo eebbinji hedhema?<br>Eet…….1 Wawwot..…..2	1 2	1 2	1 2
415	Saxxeexxa hashsha agobere giddo gale'e?<br>Eet…….1 Wawwot..…...2	1 2	1 2	1 2
416	Saxxeexxe 2 barra giddo qufa'I hedhema?<br>Eet…….1 Wawwot..…...2	1 2	1 2	1 2
**Qortuma 416 waawwot kadoole qortuma 419 sa'we**				
417	Quffa'a jamerian meaa bara?	-------------Barra	--------------Barra	---------------Barra
418	Qufa'a afeeki,edi'a irri darre yo'oshsha hafuura adhdhatixxi ko'o hexxee?<br>Eet…….1 Wawwot ..…...2	1 2	1 2	1 2
419	Wiinamik hafuura adhdhaaki mitte daqiiqa'n me'ele hafuura adhdha laakkosi	______________<br>Mitte daqiqa'n hafuura adhdhaaki laakkoosik	______________<br>Mitte daqiqa'n hafuura adhdhaaki laakkoosik	______________<br>Mitte daqiqa'n hafuura adhdhaaki laakkoosik

420	Hafuura adhdhaaki raakko honne bonchoo?<br>Qomma chall'a…………….1<br>Saanoo chall'aa.………...2<br>Leeminning boncho...................3<br>Egenebon...................4	1 2 3 4	1 2 3 4	1 2 3 4
421	Sannotika fullaakke kaalakke gashoo anno affee?<br>Eet……….1 Wawwot ………... 2<br>Egenebon.................3	1 2 3	1 2 3	1 2 3
422	Dukuba fayuuntaxxi mine meeyea?<br>Eet……..1 Waawwot…….2	1 2	1 2	1 2
**Laakkoossa 422 wawwot kaddolle laakkoossa 427 sa'we**				
423	Hoone cilotike mine'n Ci'lo habaa'n alfitette?<br>Hoosipitaala ardinke'n........1<br>Xeena xaaba mootumatike'n…2<br>Xeena keela…….3<br>Mootumatixxe kadebaaxxe orga Kilinike…......4<br>annichchinke / kilinike…….5<br>wele annichchinxe orga…....6<br>Suqee…………7<br>Qorsii mini…….8<br>Aadatixxa ci'lo….9	1 2 3 4 5 6 7 8 9	1 2 3 4 5 6 7 8 9	1 2 3 4 5 6 7 8 9
424	Anno hitteexxa yanna'n cilotika mine geesisete?	Barra----------	Barra----------	Barra----------
425	Heene kiniine adhdhitete?<br>Wele bifa kiniine hexxoole borreesse<br>Bii'netixa kiniine..........1<br>Baakiteriya balleesitaaxxa.............2<br>Foww assitaaxxa...................3<br>Wele qorsa...................4	1 2 3 4	1 2 3 4	1 2 3 4
426	Oose hittee qico ci'lotika mine mammarat adhdhiteexxi?(saxxeexxe 2 torba'n)<br>Eet……..1 Waawwot ……….2	1 2	1 2	1 2
**Qortuma 426 wawwot kaddolle ortuma 428 sa'we**				

427	Mitte-mitte ci'lotika mine mammaremat ci'lo alfitaaxxe oose'a fulaaki maallaaqi (hosipitaala/xeenaxaaba) (loolinke hira'n)<br><br>1. kardetexxi<br>2..Qorsake'e<br>3.Labaratoorete<br>4.Malchate'e<br>5. Fayyatixxe orga'a Transportetixxe woce'a<br>6. Higgeenni badadate'e<br>7.Galduminxi ma'ma barrrate<br>8. Aadatixxe ci'lo'a kaffaendeexxen<br>9.Duchinge hira	Hira<br>1.-------------------<br>2.-------------------<br>3.-------------------<br>4.-------------------<br>5.-------------------<br>6.-------------------<br>7.------------------<br>8.-------------------<br>9. -------------------	Hira<br>1.-------------------<br>2.-------------------<br>3.--------------------<br>4.--------------------<br>5.--------------------<br>6.-------------------<br>7.------------------<br>8.-------------------<br>9.--------------------	Hira<br>1.-------------------<br>2.-------------------<br>3.--------------------<br>4.--------------------<br>5.--------------------<br>6.--------------------<br>7.------------------<br>8.---------------------<br>9. ------------------
428	Anno afiteexxi ci'lonna gorsi maachcho꞉ buuto maachchoma?<br>Soodo fayyaani hineexxi Herregat.............1<br>Maalaqa gopha..........2<br>Dhibo haranga jabaxxe kadaban.............3<br>Ci'lote'n addata gopha..............4<br>La'o alfinaaxxi bakka bayyachcho..........5<br>wele hexxoole ifisse………….6	1 2 3 4 5<br>6-------------	1 2 3 4 5<br>6---------------	1 2 3 4 5<br>6-------------------
429	Saxexa 6 agenjo anno Vit A kitbatee addee?<br>Eet........1 Hedhebaan....2	1 2	1 2	1 2
430	Saxexa 6 agenjo anno godobaken chorokaen qorssa addee?<br>Eet........1 Hedhebaan....2	1 2	1 2	1 2
Codee------------------------				

**Wogga 2-59 agenjjo qico'n hexxeexxe oosseka a'urrenna hujja**				
		Annoti summi	Annoti summi	Annoti summi
	Annotixxi Codee			

431	A’urri kiio giraamet	____________<br>Kilo giraame	____________<br>Kilo giraame	____________<br>Kilo giraame
432	Hujja seenti meeteret	-----------------<br>Seenti meeter	-----------------<br>Seenti meeter	-----------------<br>Seenti meeter
433	Iimixxi ciqile mika wogga 6 agenji iima kaddeexxi oose	Iimixxi ciqile mika seenti meetiret-----------------	Iimixxi ciqile mika seenti meetiret…………..	Iimixxi ciqile mika seenti meetiret………..

Codee-------------------------

**Hafuura 5; Oosetixxa kitibaate ,fayyatikanna sagaletika jeeja**				
		Annoti summi	Annoti summi	Annoti summi
	Annotixxi Codee			
	Wogga agenjoken	------------------agenjo	----------------- agenjo	-------------agenjo
501a	Kittibatexxa oddoo laaqee egendet<br>Eet….1 Wawwot…..2	1 2	1 2	1 2
501b	Kittibatexxa oddoo habenn laqet<br>Fayyatixxi ogeeyye.......1<br>Jalloteken...........2<br>Radioxxe.........3<br>Televsionaxx.......4<br>Barachotixx mine.......5<br>Olaaken.........6	1 2 3 4 5	1 2 3 4 5	1 2 3 4 5
501c	Anno kittibate hanno jammeren<br>Elledeshan.......1<br>Mitte agenjo saxxexi..........2<br>Egenebonnon..........3	1 2 3	1 2 3	1 2 3
501d	Anno kittibate muunxxasha meyee gize adhaa hasisssan<br>Miittele..........1<br>lelmmele.........2<br>sessele..........3<br>sholeele..........4<br>Onddelee ....5<br>Egenebonnon.......6	1 2 3 4 5 6	1 2 3 4 5 6	1 2 3 4 5 6
501e	Meeyea woggan anno kittibatee birraan<br>Mitte woggan eddessa.....1<br>Mitte wogganana saaeaxxi.......2<br>Egenebonnon.......3	1 2 3	1 2 3	1 2 3

502	Amma kitibaxxa hedda			
502a	Kittibaxxi programme malaqaa hassisban<br>Kadowalle.....1<br>Kaddeqo......2	1 2	1 2	1 2
502b	Kittibatee dhukubba gibbisan<br>Kadowalle.....1<br>Kaddeqo......2	1 2	1 2	1 2
502c	Kittibatee annoaa hasisan<br>Kadowalle/.....1<br>Kaddeqo......2	1 2	1 2	1 2
502d	Kittibate assesach lommo kadeebakee dhukkuba<br>Kadowalle.....1<br>Kaddeqo......2	1 2	1 2	1 2
502e	kittibatetixxe buuton geltaaxxarakko yeosheen<br>Kadowalle.....1<br>Kaddeqo......2	1 2	1 2	1 2
502f	Kittibate anno elagopissan<br>Kadowalle.....1<br>Kaddeqo......2	1 2	1 2	1 2
503	Anno dhukkuba faachcheffataaka kittibaate alfite'en.<br>Eet……….1<br>Wawwot……..2	1 2	1 2	1 2
**Qortuma 503 wawwot kaddolle qortuma 516 sa'we**				
504	Anno a'urre leelishaaxxa kaarede afe'e?<br>Eet…….1 waawwo…...2	1 2	1 2	1 2
**Qortuma 504 wawwot kaddolle qortuma 507 sa'we**				
505	Anno saanba naqarisiinxa kittibaate adhite'e? Gadaneesi hedheexxa qulleesse<br>Eet…….1 Wawwot...2	1 2	1 2	1 2

<table>
<tr><td>506</td><td>Kitibaatetixxe kaarde’n mitte mittende’n kittibaatetixxi barra borresende’e?

Kittibaate uwwendeexxi barra borreesema gophoole wogga hitaaxxe bakka’n "44"laakkoossa Borreesse</td><td>Barra agenjo wogga
Saanba neqersa/___|______|__
Pooliyo0___|______|_
Pooliyo 1___|___|____
Pooliyo2___|______|_
Pooliyo3___|______|_
DPT1_______|______
DPT 2___|______|___
DPT3___|______|___
PCV1____|______|__
PCV2___|______|___
PCV3___|______|___
Rota1___|______|___
Rota 2___|______|___
Hiffanna___|______|_
viitamineA___|___|__</td><td>Barra agenjo wogga
Saanba neqersa/___|______|
Pooliyo0___|_____|
Pooliyo1___|___|___
Pooliyo2___|_____|
Pooliyo3___|____|
DPT1___/____|____
DPT2___|______|__
DPT3___|_____|__
PCV1____|_____|_
PCV2___|_____|__
PCV3___|___|__
Rota1___|___|___
Rota 2___|____|___
Hiffanna___|____|
viitamineA___|___|_</td><td>Barra agenjo wogga
Saanba neqersa/___|______|
Pooliyo0___|______|
Pooliyo1___|______|
Pooliyo2___|___|__/
Pooliyo3___|____|__
DPT1____|____|__
DPT2___|______|__
DPT3___|____|____|
PCV1____|______|_
PCV2___|____|___
PCV3___|______|__
Rota1___|______|__
Rota2___|______|__
Hiffanna___|____|
viitamineA___|___|_</td></tr>
<tr><td colspan="5">Ama annotixxa kittibaatetixxa kaarde affebaaxxe</td></tr>
<tr><td>507</td><td>BSGixxa kittibaate TB faachcheataaxxa yo’oxxa yanna gadaneessa leellishaaxxa adhite’e?
Eet..........1 Hedhebaan.…...2</td><td>1 2</td><td>1 2</td><td>1 2</td></tr>
<tr><td>508</td><td>Gadaneessi hedheexxa mamisaaxxe
Eet……….1 Wawwot……...2</td><td>1 2</td><td>1 2</td><td>1 2</td></tr>
<tr><td>509</td><td>Pooliyotixxa kittibaate,af’o’inxa adhite’e ?
Eet …….1 Waawwo …...2</td><td>1 2</td><td>1 2</td><td>1 2</td></tr>
<tr><td colspan="5">Qortuma 509 wawwot kaddolle qortuma 512 sa’we</td></tr>
<tr><td>510</td><td>Taakkaa woddixxi pooliyo haanote?
ilemeeshshanniዴ፧lame torba giddo ፧lame torba iimi darre
Ilemeeshshanni…………1
Lame torba iimi darre...........2</td><td>1 2</td><td>1 2</td><td>1 2</td></tr>
</table>

511	Hitteexxe yanna poliyotixxa kittibaate adhiteexxi<br>Lakkoossiki________	----------------<br>Lakkoossiki	----------------------<br>Lakkoossiki	-------------------<br>Lakkoossiki
512	DPTxxa kittibaate luqqetixxa/dolliinxa adhdhite'en<br>Eet…….1<br>Waawwot…...2	1 2	1 2	1 2
**Qortuma 512 wawwot kaddolle qortuma 514 sa'we**				
513	DPTixxa kittibaate hitteexxe yanna'a adhdhite'e?<br>Laakkoossik______	-----------------<br>Lakkoossiki	-----------------<br>Lakkoossiki	--------------<br>Lakkoossiki
514	PCV ixxa kitbaate luqqetixxa/dolliinxa adhdhite'en<br>Eet….1 Waawwot…...2	1 2	1 2	1 2
515	Albasteke uunake Rotaixxa kittibaate adhdihite'en (Affeken eguunach)<br>Eet….1 Waawwot…...2	1 2	1 2	1 2
514	Hiffanatika dhukkuba faachcheffachchote'e 9 agenjo'nuwwendaaxxi kittibaate adhite'en.<br>Eet….1 Waawwot…...2	1 2	1 2	1 2
515	Anno kittibaate birtaa wodda kittibaate muuxxate'e woraqat uwweme'een. Woraqat uwwemeeka leellishshe<br>Eet…………1<br>Hedhebaan………..2	1 2	1 2	1 2
516	Annotiki kittibatetik jeeji yookin kaardetenaa'n yookin amatenaa'n afemaaka tarja uuddaat anno haranga yookin heenooxxa kittibaate alfiteexxe kaddoole buuto laqi! mitti iimi darre kada dande'aan.	1. Kittibate muxxike<br>2. Kittibate adedhebakkh<br>3 . Kittibate gemashe adeheke<br>4.Kittibate eski/eskek woggan adheke	1. Kittibate muxxike<br>2. Kittibate adedhebakk<br>3 . Kittibate gemashe adeheke<br>4.Kittibate eski/eskek woggan adheke	1. Kittibate muxxike<br>2. Kittibate adedhebakk<br>3 . Kittibate gemashe adeheke<br>4.Kittibate eski/eskek woggan adheke
517	Anno kittibate adedhebaken ena kittibate gemashe adehek,mayekken waldene?			

	1. Kittibatetixxa hasensha ege’na gopha<br>2. Langaxxe/sakkaxxa kittibaate hexxeexxa ege’na gopha<br>3. Habanna haano kittibaate uunaaxxa ege’na gopha<br>4. kittibatetixxe buuton geltaaxxarakko gibat<br>5. Hoyyendeexxa yaane dedhiinxxe uushshon uuda<br>6. Kittibaate’n iima adde gopha<br>7. kittibaate uwwendeexxe baaka bayyata<br>8. Kittibaate uwwndaaxxei yanna eldaaxxe yanna kada gopha<br>9. Kitibaate uwwa manji gophema<br>10. Kittibaate hedha gopha<br>11. Anno elagophatenaa’n kexxeexxa kittibaate giba<br>12. Qeerraaxxa yanna heqqa	1 2 3 4 5 6 7 8 9 10 11 12	1 2 3 4 5 6 7 8 9 10 11 12	1 2 3 4 5 6 7 8 9 10 11 12
